# Comparative Outcomes Between Needle Aspiration and Incision-and-Drainage in Breast Abscesses: Is Less Truly More?

**DOI:** 10.7759/cureus.95402

**Published:** 2025-10-25

**Authors:** Bhavya Rao, Chirantan Suhrid, Akanksha Mishra, Jayashri Pandya

**Affiliations:** 1 General Surgery, Topiwala National Medical College and BYL Nair Charitable Hospital, Mumbai, IND

**Keywords:** benign breast diseases, breast abscess, incision and drainage, needle aspiration, patient satisfaction, treatment outcome

## Abstract

Background

The incidence of breast abscesses has increased in recent years, especially in developing countries. Breast abscesses have traditionally been treated with incision and drainage (InD), but there has been a shift toward USG‐guided percutaneous catheter placement or USG-guided and/or blind needle aspiration (NA). Our study aimed to determine which procedure was the best via comparison.

Methodology

We undertook this two-year prospective observational study at our tertiary care center. The study included 60 participants whose treating surgeons advised them to undergo either an NA or an InD. They were then divided into two groups of 30 patients each. Previously defined parameters of interest were observed and recorded. The data was tabulated and analyzed, with a *P*-value of 0.05 considered significant.

Results

A statistically significant correlation between four variables was identified: age of participants, size and volume of breast abscesses, recovery time post-procedure, and the procedure of choice. No statistical relevance was found in the relationship between parity status and culture sensitivity reports and the chosen interventional technique.

Conclusions

Younger women prefer opting for an NA, although recurrence rates are greater than for InD. Staphylococcus aureus was identified as the most common organism, with drug-resistant forms accounting for approximately 63% of the samples. The size of breast abscesses may play a role in intervention choice, but researchers' opinions vary. When all variables and their associations are considered, we advocate that the treating surgeon be the lead decision-maker who personalizes decisions for each patient.

## Introduction

Mastitis can either present as diffuse inflammation of the breast tissue or localize to form an abscess. Mastitis has an incidence rate ranging from 1% to 33%, and its subset, breast abscesses, occurs in about 4.6% to 11% of lactating females [1]. While abscesses are commonly seen in the lactating population, they are also seen in non-lactating females, but infrequently. While infectious etiology is more common, non-infectious causes of mastitis include periductal mastitis (a sequel to duct ectasia) and foreign body-associated inflammation (due to piercings or implants) [2].

The risk factors for developing lactational breast abscesses are a higher maternal age at delivery time, a gestation period of longer than 41 weeks, previous or current mastitis, working away from home, and anatomical considerations such as cracked nipples [3]. Other variables, such as educational status, breastfeeding combined with complementary feeds, and inverted or flat nipples, appear to aggravate disease severity [4]. Diabetes and smoking significantly increase the risk of developing non-lactational breast abscesses and periductal mastitis [5].

Breast abscesses are commonly caused by skin-colonizing bacteria, with Staphylococcus aureus (S. aureus) being the most common. S. aureus isolates appear to be methicillin-resistant, making further management cumbersome. Polymicrobial breast abscesses account for up to 40% of cases and may contain aerobic microorganisms such as Staphylococcus, Enterobacteriaceae, and Streptococcus, and anaerobic microbes such as Peptostreptococcus, Propionibacterium, and Bacteroides. The latter group of organisms is more commonly seen in smokers and diabetics. Unusual organisms that rarely trigger breast abscesses include Bartonella, Mycobacteria (both tuberculous and atypical), certain fungi (Candida and Cryptococcus), and parasites and maggots, perhaps an indication of underlying immunodeficiency conditions, such as HIV [1].

A breast abscess diagnosis is mainly clinical, but differentiating between an abscess and mastitis can be difficult. An ultrasound (USG) scan is necessary to differentiate between the two, as their management is drastically different. An abscess appears on a USG as a collection, often hypoechoic, with a thick periphery, while mastitis appears as an area with altered echotexture with hyperechogenicity in the inflamed region. Mastitis generally requires management with oral/intravenous antibiotics, preventing it from turning into an abscess, whereas an existing abscess requires surgical intervention [6].

Traditionally, breast abscesses have been treated with incision and drainage (InD), but there has been a recent shift toward drainage via either USG‐guided percutaneous catheter placement or needle aspiration (NA). If required, repeated NAs may be performed, or suction-drainage devices may be placed in the abscess cavity to drain the pus. The dilemma is clear: Is a simple NA sufficient, or do patients require more elaborate surgical intervention with InD? To address this dilemma, we undertook this study to investigate and compare these two modalities to establish the best treatment by comparing the outcomes after each modality was employed.

This article was presented as a free paper at the 84th Annual Conference of the Association of Surgeons of India (ASICON), held in Agra, India, on November 26, 2024.

## Materials and methods

This study aimed to investigate by comparison of NA and InD in treating breast abscesses. Its objective was to identify the advantages and disadvantages of each method when comparing certain parameters, as have been enumerated below, and to compare the outcomes, efficiency, and effectiveness of each method. We conducted a prospective observational study over two years (2022-2024) among patients who visited the General Surgery Outpatient Department in our tertiary care center.

After obtaining Institutional Ethics Committee approval (Ref No: ECARP/2020/155), written and informed consent were obtained from 60 female patients before collecting the prospective data. These patients were diagnosed with breast abscesses by their treating surgeons and admitted for either NA or InD treatment. Our study population included patients diagnosed with the additional inclusion criteria of an age of over 18 years. Those who did not give consent and presented with infections secondary to underlying malignant breast lesions were excluded from the sampling population. The population was then divided into two groups of 30 patients each, and the following parameters were recorded:

1. Volume and size of breast abscess according to clinical and radiological assessments

2. Method of management adopted: NA or InD

3. Number of sittings undertaken for NA

4. Level of postoperative pain

5. Period of hospital stay

6. Recurrence of breast abscess post-intervention

Post-intervention, both groups of patients were treated with injectable antibiotics (empirically treated with Amoxycillin-Clavulinic Acid and Metronidazole) and painkillers for 48 hours, following which they were started on oral antibiotics, based on their culture-sensitivity reports, and discharged. They were then put on regular follow-up for wound assessment and, if required, further intervention for a period of one month. Those that developed abscesses within this period were termed *recurrences*, and those that were free from disease (i.e., the complete absence of clinical signs and typical USG findings associated with an abscess) were taken to be part of the *recovery* group. The observations made for each group were tabulated and analyzed using SPSS version 27 (IBM Corp., Armonk, NY) software, with a *P*-value of 0.05 considered significant. No distinction was made between USG-guided aspiration and blind NA in the data tabulation.

Appropriate significance tests, such as Mann-Whitney’s and Chi-square tests, were applied to achieve the study's objectives. When the data were qualitative, numbers and percentages were calculated. For the quantitative data, means, standard deviations, and coefficients of variation were used for analysis.

## Results

The participants’ ages ranged between 19 and 49 years, with an average of 33.26 years in the InD group and 29.43 years in the NA group, with an overall mean of 31.35 ± 6.29 years. The difference was statistically significant, with *P* < 0.05 (Table 1).

**Table 1:** Distribution of age between the two groups.

Age interval (years)	Frequency, *n* (%)
0-9	0 (0)
10-19	1 (1.67)
20-29	27 (45)
30-39	29 (48.33)
40-49	3 (5)
50-59	0 (0)
N	60
Mean	31.35
Std. deviation	6.286

The mean diameter among breast abscesses that underwent InD was 5.6 cm, while that of those that underwent NA was 3.6 cm. On applying the t-test, the difference in these values was significant (*P* < 0.01).

Table 2 indicates that the smallest breast abscess in the group that underwent InD was 3 cm, and the largest that underwent NA was 5 cm. The majority of patients who underwent InD had breast abscesses with sizes greater than 5 cm. In the NA group, the majority of patients had breast abscess sizes ranging between 3 and 5 cm.

**Table 2:** Size of abscesses and procedure that was undertaken.

Size of abscesses (cm)	Incision and drainage, *n* (%)	Needle aspiration, *n* (%)
<3	2 (6.67)	4 (13.33)
3-5	13 (43.33)	24 (80)
>5	15 (50)	2 (6.67)

Statistical significance was noted when comparing the volumes of abscesses in the two groups (Table 3). The mean abscess volume was 77 cc in the InD group and 25 cc in the NA group. 

**Table 3:** Determination of the volume of the abscess between the two groups.

Groups	*N*	Mean rank	Sum of ranks	Mann-Whitney U test	*P*- value
Incision and drainage	30	43.57	1307.00	58.00	<0.01
Needle aspiration	30	17.43	523.00		

No significant association was identified between parity and the treatment option chosen (Table 4). 

**Table 4:** Distribution of parity between the two groups.

Groups	Nulliparous, *n* (%)	Primiparous, *n* (%)	Multiparous, *n* (%)	Total (*N*)	Chi-square	*P*-value
Incision and drainage	5 (16.7)	8 (26.7)	17 (56.7)	30	2.651	0.266
Needle aspiration	9 (30)	10 (33.3)	11 (36.7)	30		

Table 5 shows that the growth of contaminants (13, 61.90%) and methicillin-resistant S.aureus (MRSA) on culture sensitivity of pus was greater in the InD group, while the growth of methicillin-sensitive S. aureus (MSSA) was greater in the NA group. No growth was identified in 14 (70%) cultures in the NA group. No significant correlation was identified in the culture sensitivity studies.

**Table 5:** Determination of culture-sensitivity reports of the pus drained under each group. ^#^Methicillin-sensitive Staphylococcus aureus. ^*^Methicillin-resistant Staphylococcus aureus.

Groups	Incision and drainage, *n* (%)	Needle aspiration, *n* (%)	Total, *n* (%)	Chi-square	*P*-value
Contaminants	13 (61.90)	8 (38.10)	21 (100)	5.867	0.118
MSSA^#^	3 (42.86)	4 (57.14)	7 (100)		
MRSA^*^	8 (66.67)	4 (33.33)	12 (100)		
Sterile	6 (30)	14 (70)	20 (100)		
Total (*N*)	30	30	60		

The data in Table 6 show that the recovery time from an NA was less than that from an InD (*P* < 0.05). The mean number of days for recovery after an NA was 8.5 days compared with 9.36 days after an InD. Interestingly, a comparison of recovery times for abscesses of equal volume between the two groups showed that the mean number of recovery days was similar - 9.13 days and 9.76 days, respectively.

**Table 6:** Comparison of recovery time between the two groups.

Groups	*N*	Mean rank	Sum of ranks	Mann-Whitney U test	*P*-value
Incision and drainage	30	34.87	1046.00	319.00	0.044
Needle aspiration	30	26.13	784.00		

Approximately 27% of patients who underwent NA required a second sitting. In these cases, a significant difference in recovery times was observed in patients undergoing NA and InD, provided they had abscesses of similar volume, as shown in Table 7. The mean days for recovery required by those who underwent a second sitting (11.5 days) were greater than those required for similar volumes of abscesses after an InD (10.12 days).

**Table 7:** Mean recovery time when comparing abscesses of equal volume undergoing a second sitting of needle aspiration.

Volume (cc)	Mean recovery time (days)	
	Incision and drainage (*n*)	Needle aspiration (*n*)
20	10	10
30	6	15
50	9.5	14
60	15	7

InD showed a 100% success rate, while NA had a success rate of 93.34% (28/30). These two failed cases were ultimately treated by performing an InD with a mean recovery time of 19 days.

On histopathology, 3 (10%) of 30 participants undergoing an InD had granulomatous mastitis. However, the NA group showed no such results.

## Discussion

The first parameter analyzed was the correlation between age and the intervention method chosen for treating each breast abscess. The mean age of patients was 33.26 years in the InD group and 29.43 years in the NA group, with an overall age range of 19-49 years. These findings are consistent with most studies, including that of Ranjeesh et al., whose study population had a mean age of 30 years [7]. Most other studies found no statistically significant correlation between age and the development of breast abscesses. For example, Karvande et al. identified a mean age of 23.20 years among participants, which aligns with the age range identified in our population, but still found an insignificant correlation [8]. Conversely, in our study, a comparison between age and the intervention method was significantly correlated, implying that younger women preferred an NA over an InD (<30 years). This assertion is plausible given that NA is a less invasive technique than InD and is associated with fewer post-procedure complications.

A statistical analysis of parity and the procedure performed yielded no significant association. We found that the maximum number of patients in both groups was multiparous. In contrast, the parity distribution in the study by Karvande et al. showed that most patients were primiparous (66.7% in the NA group and 56.7% in the InD group); however, this finding was not statistically significant [8]. More multiparous subjects were diagnosed with breast abscesses in our study, a finding contradicting the popular opinion that primiparous women are more susceptible to the disease due to a lack of experience regarding positioning of the baby, nipple-areola care, and breast hygiene. The explanation for this anomalous finding in our study, although statistically insignificant, lies in the fact that multiparas are subjected to an increased cumulative breastfeeding exposure that increases with their parity. Thus, they are at a higher risk of developing milk stasis and mastitis - both known risk factors for breast abscesses. Coupled with repeated cycles of breast engorgement, involution, and remodeling that lead to changes in ductal architecture and reduced efficiency of milk drainage, their predisposition to infections causes them to outnumber their primiparous counterparts.

While correlating the size and volume of our sample of breast abscesses with the interventional procedure of choice, we found that the mean diameter of breast abscesses of patients in the InD group was 5.6 cm and that of the NA group was 3.6 cm - a statistically significant correlation indicating that InD should be performed for larger abscesses (>5 cm) and NA for smaller ones. A similar statistical significance was identified when comparing the volume of the abscess and the procedure performed, potentially justifying opting for an NA to treat smaller abscesses and an InD for larger ones. The available literature has noted that the volume of the abscess is not currently a disease-specific consideration for procedure choice. Recent studies have omitted this variable or included it only as part of the general demographic of each abscess. Earlier studies, such as that by Eryilmaz et al., noted this but did not attempt to correlate it with interventional procedures [9]. Of the few newer studies commenting on volume, Li et al. emphasized that aspiration for the drainage of abscesses of more than 50 cc must be reconsidered as the failure rate is high [10].

Conversely, the abscess size is a significant consideration in procedure choice. Studies undertaken in multiple centers worldwide have advocated that NA be used for smaller abscesses and InD be reserved for larger abscesses. The opposing view, which is also noted in much of the recent literature, suggests that abscess size may not play a role in procedure choice. Colin et al.’s French study concluded that USG-guided aspiration is effective for abscesses larger than 5 cm. Similarly, David et al.’s study recommends de-emphasizing size, as NA should always be attempted first [11,12]. In fact, Fardhus et al. concluded that NA must be the first line of treatment and is effective in abscesses as large as 7 cm [13].

The next parameter that we analyzed was the association between culture sensitivity studies and the interventional method of choice. Our study identified no statistically significant association between the culture sensitivity studies of the two groups. Compared with existing reportage, organisms isolated from specimens taken after an NA appear similar. For example, in Ding et al.’s study, 176 of 215 samples were identified as infected with S. aureus, either MSSA or MRSA [14]. Similarly, extensive research has identified S. aureus as the most common organism isolated from samples obtained by NA. Some studies have also identified S. aureus as the most common organism, irrespective of the drainage method. For example, Totadri et al. found that 40% of isolates were S. aureus, with a further 6% being MRSA [15]. S. aureus is the most common organism implicated in forming breast abscesses, likely because it is the most common skin commensal to cause an infection of superficial skin or mucosal layers exposed to the environment. Of greater concern is the increasing incidence of MRSA, suggesting that the widespread use of antibiotics has contributed to the emergence of these drug-resistant strains in the development of infection.

In the context of infections, another condition worth mentioning is granulomatous mastitis. It typically presents as a palpable mass often accompanied by overlying skin and nipple changes that make differentiation from an underlying malignancy challenging. In approximately 15% of patients with a breast lump, the mass may clinically mimic an abscess or present as axillary lymphadenopathy. The lack of specificity of symptoms confounds the diagnostic process, creating the illusion that the interventional procedure is inappropriate. These patients may initially undergo abscess puncture, drainage, incision, or lump excision. However, as aspiration can fail to diagnose the disease and a sample of tissue is required for diagnosis, using NA for all breast abscesses is questionable [16]. The prevalence of granulomatous mastitis and the frequency of its misdiagnosis must be studied further to identify the appropriate course of action.

Our study identified a 26.66% (8/30) recurrence rate in the NA group, of which six participants recovered after a second sitting. The remaining two participants underwent a third sitting of NA; however, due to another recurrence post-procedure, InD was performed. Most studies in the existing literature have reported varying success rates for NA, ranging from 73.5% in Sushel et al.’s study [17] to 96% in Colin et al.’s study [11], while the success rate for InD patients is typically 100%. 

Fathy et al.’s study identified two recurrences in the USG-guided aspiration group (11.8%) and no recurrences among InD patients during a one-month follow-up period. Interestingly, in this same study, the persistence of signs and symptoms - regardless of the procedure and even after a second intervention attempt lasting more than two weeks - was classified as a *treatment failure*. USG-guided aspiration showed a failure rate of 29.2% (7/24), while there were no failures following InD treatment [18]. Javed et al. found seven recurrences (23.33%) in Group A (InD) and 21 (70.0%) in Group B (multiple NA). The identified statistical significance implied that the likelihood of failure is higher with NA. This study also showed that post-InD recurrences are possible, which contradicts the consensus on post-InD recurrence [19]. Gandhi et al. found that among 130 participants, there were no recurrences in the USG-guided NA group and 3.1% post-InD recurrences [20]. The inference is that the likelihood of recurrence is higher following NA, due to factors such as incomplete drainage, inadequate or insensitive antibiotic coverage, and the presence of underlying mastitis. Post-InD recurrences are less likely because adequate pus drainage and repeated dressings prevent persistent infection in the abscess cavity.

In the present study, recovery after intervention occurred less often in the NA group. Recovery was considered to be a complete resolution of the abscess with no clinical/radiological evidence in the follow-up period. It may be argued that a one-month follow-up period is insufficient to capture all recurrences, which could lead to generalizations that may not necessarily apply to the broader population. Upon reviewing the literature, we found that the vast majority of studies had follow-up periods similar to those of Fathy et al. and Gandhi et al., both of which followed patients for one month [18,20]. In fact, some studies followed patients for a much shorter duration; for example, Javed et al. followed patients for only seven days [19]. Our one-month follow-up period was chosen after carefully reviewing the existing literature and considering the patients’ ability to adhere to longer-term follow-up care. Considering that they invariably come from far-flung areas, the arrangements that need to be made are monetarily detrimental to them, and longer follow-up periods would result in attrition. Another reason why we restricted this period to one month is that abscesses that occur after this time frame could be a completely new process, not necessarily related to the previous abscess.

The mean recovery time (in days) after NA was 8.5, and 9.36 after InD. However, the mean recovery time for those undergoing a second sitting of NA was 11.5 days, which was longer than that for similar-volume abscesses after InD, 10.12 days. Our findings align with the consensus that recovery is faster when NA is performed. For example, in Ranjeesh et al.’s study, the mean healing duration was 19.20 days in the NA group and 30.17 days in the InD group, respectively [7]. Fathy et al. also reported a faster recovery time for the NA group compared with the InD group: 11.16 ± 2.01 days versus 21 ± 3.12 days, respectively [18]. However, some studies have contested this finding, postulating that healing and recovery times are not statistically related to the type of procedure undertaken. For example, Gandhi et al.’s study established that the healing rate does not depend on the treatment method. The explanation for this assertion is that pus removal is the only factor affecting recovery time, and the method chosen for this removal is immaterial [20].

Our study relied on the experience of the treating surgeon in managing breast abscesses, which can be considered a limitation, as it introduces an element of subjectivity, essentially leading to selection bias. Another limitation is that NA and InD cannot be compared because there are indications to assign patients for management via NA or InD. Our study considered these fallacies and attempted to eliminate them by designing this study to investigate whether the advised treatment plan was prudent and simultaneously assimilated the patients’ response to each treatment method. Nonetheless, these limitations are inherent in the study design we envisaged.

## Conclusions

Protocol dictates that NA be attempted twice before a patient is offered InD. Our study determined that such a protocol, although implemented worldwide, may not be entrenched in decisive reportage on this subject. Multiple studies contest the notion of NA being a better mode of management among the variables that have been considered, supposedly the ones that define a superior management technique. However, we argue that the treating surgeon best determines the choice of procedure after considering the available options and that such a decision must be made after consulting with the patient and their relatives.
